# Association of Estimated Cardiorespiratory Fitness in Midlife With Cardiometabolic Outcomes and Mortality

**DOI:** 10.1001/jamanetworkopen.2021.31284

**Published:** 2021-10-29

**Authors:** Joowon Lee, Rebecca J. Song, Ibrahim Musa Yola, Tara A. Shrout, Gary F. Mitchell, Ramachandran S. Vasan, Vanessa Xanthakis

**Affiliations:** 1 Section of Preventive Medicine and Epidemiology, Boston University School of Medicine, Boston, Massachusetts; 2 Department of Epidemiology, Boston University School of Public Health, Boston, Massachusetts; 3 Residency Program, Department of Internal Medicine, Boston Medical Center, Boston, Massachusetts; 4 Cardiovascular Engineering, Inc, Norwood, Massachusetts; 5 Section of Cardiovascular Medicine, Department of Medicine, Boston University School of Medicine, Boston, Massachusetts; 6 Center for Computing and Data Sciences, Boston University, Boston, Massachusetts; 7 Framingham Heart Study, Framingham, Massachusetts; 8 Department of Biostatistics, Boston University School of Public Health, Boston, Massachusetts

## Abstract

**Importance:**

The associations of estimated cardiorespiratory fitness (eCRF) during midlife with subclinical atherosclerosis, arterial stiffness, incident cardiometabolic disease, and mortality are not well understood.

**Objective:**

To examine associations of midlife eCRF with subclinical atherosclerosis, arterial stiffness, incident cardiometabolic disease, and mortality.

**Design, Setting, and Participants:**

This cohort study included 2962 participants in the Framingham Study Second Generation (conducted between 1979 and 2001). Data were analyzed from January 2020 to June 2020.

**Exposures:**

eCRF was calculated using sex-specific algorithms (including age, body mass index, waist circumference, physical activity, resting heart rate, and smoking) and was categorized as: (1) tertiles of standardized eCRF at examination cycle 7 (1998 to 2001); (2) tertiles of standardized average eCRF between examination cycles 2 and 7 (1979 to 2001); and (3) eCRF trajectories between examination cycles 2 and 7, with the lowest tertile or trajectory (ie, low eCRF) as referent group.

**Main Outcomes and Measures:**

Subclinical atherosclerosis (carotid intima-media thickness [CIMT], coronary artery calcium [CAC] score); arterial stiffness (carotid-femoral pulse wave velocity [−1000/CFPWV]); incident hypertension, diabetes, chronic kidney disease (CKD), cardiovascular disease (CVD), and mortality after examination cycle 7.

**Results:**

A total of 2962 participants were included in this cohort study (mean [SD] age, 61.5 [9.2] years; 1562 [52.7%] women). The number of events or participants at risk after examination cycle 7 (at a mean follow-up of 15 years) was 728 of 1506 for hypertension, 214 of 2268 for diabetes, 439 of 2343 for CKD, 500 of 2608 for CVD, and 770 of 2962 for mortality. Compared with the low eCRF reference value, high single examination eCRF was associated with lower CFPWV (β [SE], −11.13 [1.33] ms/m) and CIMT (β [SE], −0.12 [0.05] mm), and lower risk of hypertension (hazard ratio [HR], 0.63; 95% CI, 0.46-0.85), diabetes (HR, 0.38; 95% CI, 0.23-0.62), and CVD (HR, 0.71; 95% CI, 0.53-0.95), although it was not associated with CKD or mortality. Similarly, compared with the low eCRF reference, high eCRF trajectories and mean eCRF were associated with lower CFPWV (β [SE], −11.85 [1.89] ms/m and −10.36 [1.54] ms/m), CIMT (β [SE], −0.19 [0.06] mm and −0.15 [0.05] mm), CAC scores (β [SE], −0.67 [0.25] AU and −0.63 [0.20] AU), and lower risk of hypertension (HR, 0.54; 95% CI, 0.34-0.87 and HR, 0.48; 95% CI, 0.34-0.68), diabetes (HR, 0.27; 95% CI, 0.15-0.48 and HR, 0.31; 95% CI, 0.18-0.54), CKD (HR, 0.63; 95% CI, 0.40-0.97 and HR, 0.64; 95% CI, 0.44-0.94), and CVD (HR, 0.46; 95% CI, 0.31-0.68 and HR, 0.43; 95% CI, 0.30-0.60). Compared with the reference value, a high eCRF trajectory was associated with lower risk of mortality (HR, 0.69; 95% CI, 0.50-0.95).

**Conclusions and Relevance:**

In this cohort study, higher midlife eCRF was associated with lower burdens of subclinical atherosclerosis and vascular stiffness, and with a lower risk of hypertension, diabetes, chronic kidney disease, cardiovascular disease, and mortality. These findings suggest that midlife eCRF may serve as a prognostic marker for subclinical atherosclerosis, arterial stiffness, cardiometabolic health, and mortality in later life.

## Key Points

**Question:**

Is estimated cardiorespiratory fitness (eCRF) in midlife associated with subclinical atherosclerosis, vascular stiffness, and risk of cardiometabolic disease and mortality?

**Findings:**

In this cohort study of 2962 Framingham Offspring Study participants, higher midlife eCRF was associated with lower burdens of subclinical atherosclerosis and vascular stiffness, and with a lower risk of hypertension, diabetes, chronic kidney disease, cardiovascular disease, and mortality over a mean follow-up of 15 years.

**Meaning:**

These findings suggest that prognostic ability of midlife eCRF may extend to a wide range of cardiometabolic diseases.

## Teaser

This cohort study examines the association of estimated cardiorespiratory fitness in midlife with subclinical atherosclerosis, arterial stiffness, incident cardiometabolic disease, and mortality among participants in the Framingham Offspring Study.

## Introduction

Cardiorespiratory fitness (CRF) is inversely associated with risk of cardiovascular disease (CVD) and all-cause mortality incrementally over established CVD risk factors.^1,2,3,4,5,6^ Additionally, the use of CRF improves CVD and mortality risk predictions when used in conjunction with established risk factors.^7,8^ Recent scientific statements from the American Heart Association emphasize the importance of assessing CRF in clinical practice.^9^

CRF is measured via cardiopulmonary exercise testing; however, this method requires in-person assessment with specialized equipment and trained personnel, rendering it expensive and less accessible.^9^ Due to such limitations, nonexercise estimated CRF (eCRF) algorithms have been developed using readily available clinical information, such as age, sex, waist circumference, resting heart rate, and physical activity. Studies have demonstrated that the prognostic ability of eCRF for CVD risk and mortality is comparable to the use of traditional CRF testing.^10,11^ However, little is known about the associations of eCRF during midlife with the prevalence of subclinical atherosclerosis and arterial stiffness, and with the development of cardiometabolic diseases and mortality later in life.

Accordingly, using data from the Framingham Offspring Study community-based sample, we examined associations of midlife eCRF with indices of subclinical atherosclerosis and arterial stiffness, and to incident hypertension, diabetes, chronic kidney disease (CKD), CVD, and mortality in later life. We hypothesized that higher midlife eCRF is associated with lower burden of subclinical atherosclerosis and arterial stiffness and a lower risk of cardiometabolic diseases and all-cause mortality compared with low midlife eCRF.

## Methods

### Study Sample

**Figure 1.**
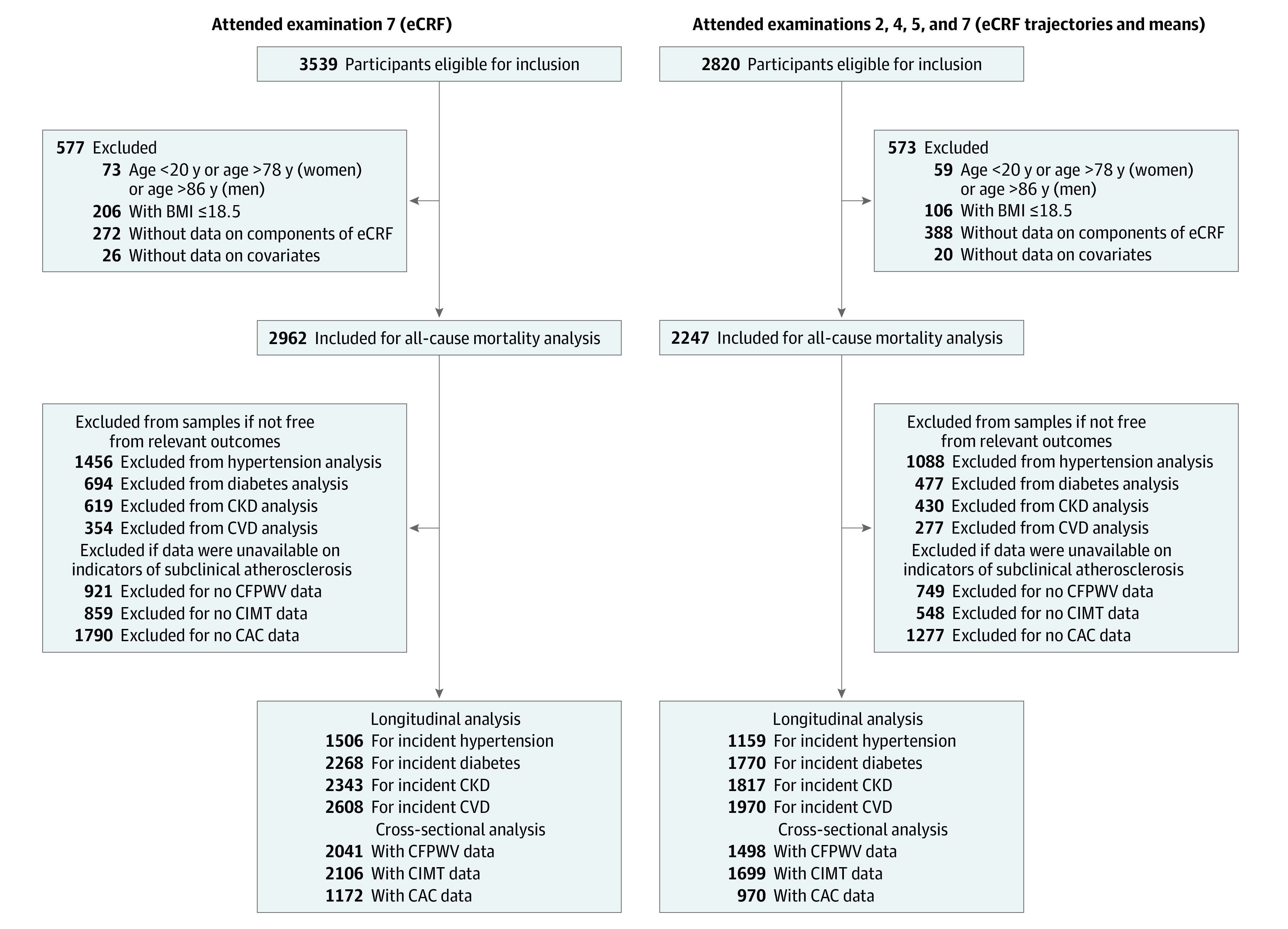
Diagram of Participant Flow BMI indicates body mass index (calculated as weight in kilograms divided by height in meters squared); CAC, coronary artery calcium; CFPWV, carotid-femoral pulse wave velocity; CKD, chronic kidney disease; CIMT, carotid intima-media thickness; CVD, cardiovascular disease; eCRF, estimated cardiorespiratory fitness. Samples for single examination eCRF (cycle 7) were numbered as follows: hypertension, sample 1.2; diabetes, sample 1.3; CKD, sample 1.4; CVD, sample 1.5; CFPWV, sample 1.6; CIMT, sample 1.7; and CAC, sample 1.8. Samples for eCRF trajectories and mean eCRF (cycles 2, 4, 5, and 7) were numbered as follows: hypertension, sample 2.2; diabetes, sample 2.3; CKD, sample 2.4; CVD, sample 2.5; CFPWV, sample 2.6; CIMT, sample 2.7; and CAC, sample 2.8.

The study design and sampling methods of the Framingham Offspring Study have been described.^12^ For the present investigation, study participants who attended examination cycle 7 (3539 participants; samples 1.1 through 1.8) and those who attended examination cycles 2 (1979 to 1983), 4 (1987 to 1991), 5 (1991 to 1995), and 7 (1998 to 2001) (2820 participants; samples 2.1 through 2.8) were eligible for inclusion, thus evaluating participants during midlife (Figure 1). Data on some components of the eCRF algorithm (eg, physical activity) were not available at examinations 3 and 6. Detailed descriptions of the study samples are in eMethods in the Supplement. The study was approved by the Boston University Medical Center institutional review board. All participants provided written informed consent. This study followed the Strengthening the Reporting of Observational Studies in Epidemiology (STROBE) reporting guideline.

### eCRF Assessment

Information on components of the eCRF algorithm was collected using a medical history questionnaire and physical examination (eMethods in the Supplement). Sex-specific longitudinal eCRF algorithms were used to assess eCRF as metabolic equivalents (METs) during midlife at examinations 2, 4, 5, and 7 via the following published sex-specific formulae^13^: eCRF in women (METs) = 14.7873 + (age × 0.1159) – (age^2^ × 0.0017) – (BMI × 0.1534) – (waist circumference × 0.0088) – (resting heart rate × 0.364) + (physical activity [active vs inactive] × 0.5987) – (smoking [yes vs no] × 0.2994), where BMI indicates body mass index (calculated as weight in kilograms divided by height in meters squared); eCRF in men (METs) = 21.2870 + (age × 0.1654) – (age^2^ × 0.0023) – (BMI × 0.2318) – (waist circumference × 0.0337) – (resting heart rate × 0.0390) + (physical activity [active vs inactive] × 0.6351) – (smoking [yes vs no] × 0.4263).

For this investigation, midlife eCRF was defined in 3 different ways: (1) single examination eCRF, defined as sex-specific tertiles of standardized eCRF at examination 7 (mean [SD], 0 [1]); (2) eCRF trajectories, defined as sex-specific eCRF trajectories between examinations 2 and 7; and (3) mean eCRF, defined as sex-specific tertiles of standardized average eCRF between examinations 2 and 7 (mean [SD], 0 [1]). For each, low eCRF was defined as the lowest tertile or trajectory group and used as referent group for analyses.

### Indices of Arterial Stiffness and Subclinical Atherosclerosis

We assessed carotid-femoral pulse wave velocity (CFPWV) as a measure of arterial stiffness, and coronary artery calcium (CAC) score and carotid intima-media thickness (CIMT) as measures of subclinical atherosclerosis. Detailed protocols for each are described in eMethods in the Supplement. In brief, to assess CFPWV, applanation tonometry was performed in 2640 participants at examination 7 via methods previously described.^14^ To quantify the CAC score, participants underwent coronary computed tomography angiography on an 8-slice multidetector computed tomography scanner (General Electric) during examination cycle 7.^15^ Lastly, CIMT was measured via carotid ultrasound by a certified sonographer following a standardized protocol at examination 8.^16,17^

### Outcomes of Interest

The outcomes of interest for this investigation were incident hypertension, diabetes, CKD, CVD, and all-cause mortality occurring after examination 7. Information on outcomes was obtained by a combination of medical history questionnaires, physical examinations at the Framingham Heart Study, hospitalization records, and communication with physicians. Hypertension was defined as systolic (SBP) of 140 mm Hg or higher, diastolic BP (DBP) of 90 mm Hg or higher, or the use of antihypertensive medication.^18^ Diabetes was defined as a fasting glucose level of 126 mg/dL or higher, nonfasting glucose level of 200 mg/dL or higher, or the use of hypoglycemic medications. CKD was defined as an eGFR of 60 mL/min/1.73 m^2^ or below.^19^ CVD was defined as the occurrence of any of the following: new-onset coronary heart disease (fatal or nonfatal myocardial infarction, unstable angina), peripheral vascular disease (symptoms of intermittent claudication), cerebrovascular disease (fatal or non-fatal ischemic or hemorrhagic stroke, or transient ischemic attack), or occurrence of heart failure. All CVD events were adjudicated by 3 physicians using standardized outcome definitions described previously.^20^ Finally, all-cause mortality was defined as death due to any cause. For CKD, diabetes, and hypertension, maximum follow-up was through examination 9 (2011 to 2014), while follow-up for CVD and mortality was through December 31, 2016.

### Covariates

Data on covariates were collected at each FOS visit from routine medical history updates (eg, age, sex, antihypertensive and lipid-lowering medication use), physical examination (SBP and DBP), and laboratory assessment (fasting plasma glucose for diabetes assessment, total cholesterol [TC], high-density lipoprotein [HDL] cholesterol) (eMethods in the Supplement). Race and ethnicity were not included as covariates because FOS participants were non-Hispanic White. The covariates were selected based on previous reports on their association with the outcomes of interest in our investigation.

### Statistical Analysis

Age- and sex-adjusted Spearman correlations were estimated between eCRF measures and variables comprising the eCRF algorithms. Group-based modeling was performed to identify subgroups of participants with a similar trajectory of eCRF (eMethods in the Supplement). CFPWV was inverse-transformed because of heteroscedasticity and multiplied by −1000 to convert units to milliseconds per meter and restore directionality. The *z* score of the mean of the maximum common carotid artery IMT and internal carotid artery IMT was obtained, and both *z* scores were averaged to generate an overall CIMT *z* score. The CAC score was natural logarithmically transformed after the addition of 1 (ln[CAC + 1]) because of its skewed distribution. We related midlife eCRF measures (ie, independent variables [single examination eCRF, eCRF trajectories, and mean eCRF] with a separate model for each) with indicators of arterial stiffness and subclinical atherosclerosis (dependent variables [CFPWV, CIMT, and CAC] with a separate model for each) using multivariable-adjusted linear regression, adjusting for age, sex, SBP, DBP, antihypertensive medication use, diabetes, TC to HDL-C ratio, lipid-lowering medication use, and prevalent CVD. We conducted sensitivity analyses excluding participants with prevalent CVD to address potential influence of prevalent CVD on associations between midlife eCRF and subclinical atherosclerosis.

To examine the associations of midlife eCRF (ie, independent variables [single examination eCRF, eCRF trajectories, and mean eCRF], with a separate model for each) with incident hypertension, diabetes, and CKD, we used Cox proportional hazards regression with discrete time intervals, adjusting for age, sex, SBP, DBP, antihypertensive medication use, diabetes, TC to HDL-C ratio, lipid-lowering medication use, and prevalent CVD. We also examined the associations of midlife eCRF (single examination eCRF, eCRF trajectories, and mean eCRF) with incident CVD and all-cause mortality using Cox proportional hazards regression, adjusting for the same covariates except for prevalent CVD in the model evaluating the association of eCRF with incident CVD, since participants with prevalent CVD were previously excluded. In sensitivity analyses, we excluded participants on antihypertensive treatment at baseline to remove any direct effects of antihypertensive medications on eCRF. We also calculated the eCRF slope as follows: (eCRF at examination cycle 7 – eCRF at examination cycle 2) / (date of examination cycle 7 – date of examination cycle 2). We then evaluated the association between eCRF slope and all outcomes. Furthermore, we evaluated the association between midlife eCRF and incident hypertension using current AHA/ACC criteria for hypertension (ie, SBP ≥ 130 mm Hg, DBP ≥ 80 mm Hg, or use of antihypertensive medication).^21^ The proportional hazards assumption was met for all models. The discriminative ability of eCRF (beyond that of standard risk factors) for all outcomes was assessed by calculating the area under the curve for receiver operating characteristic curves (Harrell C statistic). A 2-sided value of *P* < .05 was considered statistically significant for all models. All analyses were performed using SAS software version 9.4 (SAS Institute Inc).

## Results

Baseline characteristics of the largest study sample (sample 1.1, 2962 participants), stratified by eCRF tertiles at examination 7 are summarized in Table 1. The mean (SD) age was 61.5 (9.2) years and 1562 (52.7%) were women. In general, participants with low eCRF were older and had a greater burden of subclinical atherosclerosis and arterial stiffness. (Trajectories of midlife eCRF are shown in eFigure, and a comparison of characteristics between participants who were included and excluded from analysis are available in eTable 1 in the Supplement.) Overall, participants excluded from the analysis were older and had a higher burden of cardiometabolic disease. We observed strong age- and sex-adjusted Spearman correlations of midlife eCRF with BMI and waist circumference (eTable 2 in the Supplement).

**Table 1.** Characteristics of Participants by Tertiles of Single Examination eCRF^a^

Variable	Participants, No. (%)		
	Low eCRF tertile (n = 961)^b^	Moderate eCRF tertile (n = 1003)^b^	High eCRF tertile (n = 998)^b^
Age, mean (SD), y	66.2 (8.6)	61.3 (7.6)	54.2 (6.9)
Sex			
Women	499 (51.9)	533 (53.1)	530 (53.1)
Men	462 (48.1)	470 (46.9)	468 (46.9)
BMI, mean (SD)	32.5 (5.7)	27.7 (3.2)	24.5 (2.7)
Waist circumference, mean (SD), cm	111.8 (13.0)	99.3 (9.1)	89.4 (9.1)
Resting heart rate, mean (SD), bpm	69 (11)	65 (10)	61 (9)
Physical activity index, mean (SD)	36.7 (6.1)	38.3 (6.7)	38.9 (6.3)
Current smoking	111 (11.6)	127 (12.7)	131 (13.1)
Blood pressure, mean (SD), mm Hg			
Systolic	134 (19)	128 (18)	119 (16)
Diastolic	74 (10)	75 (10)	73 (9)
Hypertension	608 (63.3)	475 (47.4)	225 (22.6)
Antihypertensive medication use	463 (48.2)	352 (35.1)	157 (15.7)
Fasting glucose, mean (SD), mg/dL	113 (31)	104 (25)	95 (12)
Diabetes	207 (21.5)	91 (9.1)	22 (2.2)
Diabetes medication use	126 (13.1)	49 (4.9)	13 (1.3)
Total serum cholesterol, mean (SD), mg/dL	197 (38)	205 (37)	199 (35)
HDL cholesterol, mean (SD), mg/dL	50 (15)	53 (16)	58 (17)
Lipid-lowering medication use	281 (29.2)	225 (22.4)	97 (9.7)
eGFR, mean (SD), mL/min/1.73 m^2^	77.8 (17.3)	83.0 (15.7)	90.2 (13.5)
Carotid artery IMT, mean (SD), mm^c^			
Internal	2.7 (1.2)	2.4 (1.1)	2.0 (1.0)
Common	0.8 (0.2)	0.7 (0.2)	0.6 (0.1)
CFPWV, mean (SD), ms/s	12.2 (3.8)	10.2 (3.3)	8.1 (1.7)
CAC, median (Q1-Q3), AU	119.7 (9.6-461.6)	47.1 (0-263.4)	1.4 (0-93.7)
Prevalent CKD	146 (15.2)	77 (7.7)	19 (1.9)
Prevalent CVD	177 (18.4)	121 (12.1)	56 (5.6)
eCRF, mean (SD), METs	8.7 (1.3)	10.3 (1.2)	11.5 (1.5)

Abbreviations: AU, Agatston unit; BMI, body mass index (calculated as weight in kilograms divided by height in meters squared); bpm, beats per minute; CAC, coronary artery calcium; CFPWV, carotid-femoral pulse wave velocity; CKD, chronic kidney disease; CVD, cardiovascular disease; eCRF, non-exercise estimated cardiorespiratory fitness; eGFR, estimated glomerular filtration rate; HDL, high-density lipoprotein; IMT, intima-media thickness; METs, metabolic equivalent of tasks.

^a^ Single examination eCRF was the largest sample of participants analyzed.

^b^ Low single examination eCRF was defined as the first tertile of eCRF at the seventh examination; moderate eCRF was defined as the second tertile of eCRF at the seventh examination; high eCRF was defined as the third tertile of eCRF at the seventh examination.

^c^ Common and internal carotid artery IMT were measured at the eighth examination cycle.

### Associations of Midlife eCRF With Indices of Arterial Stiffness and Subclinical Atherosclerosis

The associations of midlife eCRF with indices of subclinical atherosclerosis and arterial stiffness are presented in Table 2. Adjusting for age, sex, SBP, DBP, antihypertensive medication, diabetes, TC to HDL-C ratio, lipid-lowering medication, and prevalent CVD, we observed that high single examination eCRF was associated with lower CFPWV and CIMT compared with the low eCRF reference (β [SE]: CFPWV, −11.13 [1.33] ms/m; CIMT, −0.12 [0.05] mm). Moreover, higher single examination eCRF was associated with lower CFPWV, CIMT, and CAC score (β [SE] per 1-SD increment: CFPWV, −5.50 [0.60] ms/m; CIMT, −0.07 [0.02] mm; CAC, −0.16 [0.08] AU). Similarly, a high eCRF trajectory was associated with lower CFPWV, CIMT, and CAC score compared with the low eCRF reference (β [SE]: CFPWV, −11.85 [1.89] ms/m; CIMT, −0.19 [0.06] mm; CAC, −0.67 [0.25] AU), and high mean eCRF was associated with lower CFPWV, CIMT, and CAC score compared with the low mean eCRF reference (CFPWV, −10.36 [1.54] ms/m; CIMT, −0.15 [0.05] mm; CAC, −0.63 [0.20] AU). Finally, higher mean eCRF was associated with lower CFPWV, CIMT, and CAC score (β [SE] per 1-SD increment: CFPWV, −5.26 [0.69] ms/m; CIMT, −0.08 [0.02] mm; CAC, −0.28 [0.09] AU). All associations remained statistically significant after excluding participants with prevalent CVD, except for the association between moderate mean eCRF and CAC (eTable 3 in the Supplement). In sensitivity analyses, we observed similar results when including the eCRF slope in multivariable-adjusted models (eTable 7 in the Supplement).

**Table 2.** Association of Midlife eCRF With Indices of Subclinical Atherosclerosis

eCRF measure^a^	Single examination eCRF			eCRF trajectories			Mean eCRF		
	No.	β estimate (SE)	*P* value	No.	β estimate (SE)	*P* value	No.	β estimate (SE)	*P* value
−**1000/CFPWV, ms/m**									
eCRF per 1-SD increment^b^	2041	−5.50 (0.60)	<.001	1498	NR		1498	−5.26 (0.69)	<.001
eCRF									
Low	573	[Reference]		208	[Reference]		427	[Reference]	
Moderate	710	−5.46 (1.10)	<.001	704	−5.39 (1.57)	<.001	521	−5.60 (1.28)	<.001
High	758	−11.13 (1.33)	<.001	586	−11.85 (1.89)	<.001	550	−10.36 (1.54)	<.001
**CIMT, mm**									
eCRF per 1-SD increment^b^	2106	−0.07 (0.02)	<.001	1699	NR		1699	−0.08 (0.02)	<.001
eCRF									
Low	605	[Reference]		247	[Reference]		486	[Reference]	
Moderate	737	−0.03 (0.04)	.42	784	−0.10 (0.05)	.06	587	−0.07 (0.04)	.09
High	764	−0.12 (0.05)	.01	668	−0.19 (0.06)	.003	626	−0.15 (0.05)	.003
**CAC, AU**									
eCRF per 1-SD increment^b^	1172	−0.16 (0.08)	.045	970	NR		970	−0.28 (0.09)	.001
eCRF									
Low	324	[Reference]		121	[Reference]		242	[Reference]	
Moderate	413	−0.09 (0.16)	.58	438	−0.31 (0.22)	.17	345	−0.41 (0.18)	.02
High	435	−0.24 (0.19)	.20	411	−0.67 (0.25)	.008	383	−0.63 (0.20)	.002

Abbreviations: AU, Agatston units; CAC, coronary artery calcium; CFPWV, carotid-femoral pulse wave velocity; CIMT, carotid intima-media thickness; eCRF, nonexercise estimated cardiorespiratory fitness; NR, not reported.

^a^ Models were adjusted for age, sex, systolic blood pressure, diastolic blood pressure, antihypertensive medication, diabetes, total cholesterol/high-density lipoprotein cholesterol, lipid-lowering medication, and prevalence of CVD at exam 7. High eCRF was defined as the highest tertile or trajectory of eCRF measures; moderate eCRF was defined as moderate tertile or trajectory of eCRF measures; low eCRF was defined as lowest tertile or trajectory of eCRF measures.

^b^ SDs are equal to 2.0 MET for all models.

### Associations of Midlife eCRF With Incident Cardiometabolic Outcomes and All-Cause Mortality

**Figure 2.**
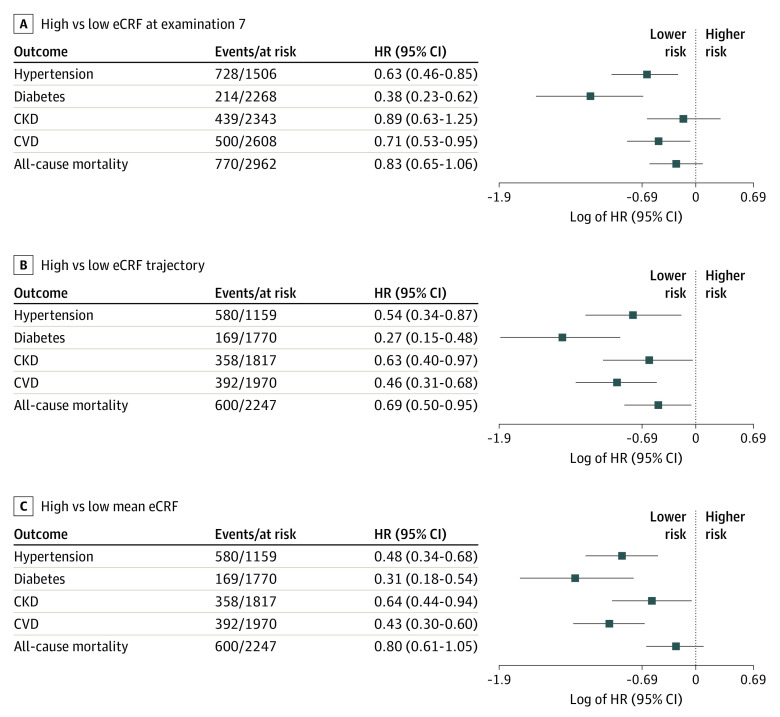
Associations of Midlife eCRF With the Incidence of Cardiometabolic Diseases and All-Cause Mortality CKD indicates chronic kidney disease; CVD, cardiovascular disease; eCRF, estimated cardiorespiratory fitness; HR, hazard ratio.

Compared with low eCRF, high single examination eCRF was associated with lower risk of developing hypertension (hazard ratio [HR], 0.63; 95% CI, 0.46-0.85), diabetes (HR, 0.38; 95% CI, 0.23-0.62), and CVD (HR, 0.71; 95% CI, 0.53-0.95) (Figure 2). Moreover, higher single examination eCRF was associated with lower risk of hypertension, diabetes, CVD, and all-cause mortality on follow-up (eTable 4 in the Supplement).

High eCRF trajectories and high mean eCRF were associated with lower risk of hypertension (HR, 0.54; 95% CI, 0.34-0.87 and HR, 0.48; 95% CI, 0.34-0.68), diabetes (HR, 0.27; 95% CI, 0.15-0.48 and HR, 0.31; 95% CI, 0.18-0.54), CKD (HR, 0.63; 95% CI, 0.40-0.97 and HR, 0.64; 95% CI, 0.44-0.94), and CVD (HR, 0.46; 95% CI, 0.31-0.68 and HR, 0.43; 95% CI, 0.30-0.60) compared with low eCRF. High eCRF trajectories were also associated with lower risk of all-cause mortality (HR, 0.69; 95% CI, 0.50-0.95) (Figure 2). We did not observe a statistically significant association between high mean eCRF and all-cause mortality; however, we observed lower risk of all-cause mortality with moderate eCRF in all 3 measures (eTable 4 in the Supplement). All associations remained statistically significant after excluding participants on antihypertensive treatment, except for the association of eCRF trajectories with incident CKD (eTable 5 in the Supplement). The associations between midlife eCRF measures and incident hypertension were attenuated when current AHA/ACC guidelines for hypertension were applied (eTable 6 in the Supplement). In sensitivity analyses, we observed similar results for outcomes when including the eCRF slope in multivariable-adjusted models (eTable 7 in the Supplement). Of note, we calculated the eCRF for men and women defined by Nes et al^11^ and examined associations with cardiometabolic and mortality outcomes; results were similar to our original results (eTable 8 in the Supplement). Overall, midlife eCRF minimally improved the discriminative ability for CVD and all-cause mortality when added to models including established CVD risk factors (Table 3).

**Table 3.** Area Under the Curve for the eCRF Algorithm

Measures^a^	Area under the curve (95% CI)				
	Hypertension	Diabetes	CKD	CVD	All-cause mortality
**Single examination eCRF**					
Traditional risk factors	0.76 (0.74-0.78)	0.88 (0.86-0.90)	0.81 (0.79-0.83)	0.69 (0.67-0.72)	0.70 (0.68-0.72)
Traditional risk factors + eCRF	0.77 (0.75-0.79)	0.88 (0.86-0.90)	0.82 (0.80-0.84)	0.71 (0.68-0.73)	0.73 (0.71-0.75)
**eCRF trajectories**					
Traditional risk factors	0.75 (0.73-0.78)	0.88 (0.86-0.90)	0.81 (0.79-0.83)	0.69 (0.67-0.72)	0.70 (0.68-0.72)
Traditional risk factors + eCRF	0.76 (0.74-0.79)	0.88 (0.86-0.91)	0.82 (0.80-0.84)	0.71 (0.69-0.74)	0.72 (0.70-0.74)
**Mean eCRF**					
Traditional risk factors	0.75 (0.73-0.78)	0.88 (0.86-0.90)	0.81 (0.79-0.83)	0.69 (0.67-0.72)	0.70 (0.68-0.72)
Traditional risk factors + eCRF	0.76 (0.74-0.79)	0.88 (0.86-0.91)	0.82 (0.79-0.84)	0.72 (0.69-0.74)	0.73 (0.71-0.75)

Abbreviations: CKD, chronic kidney disease; CVD, cardiovascular disease; eCRF, nonexercise estimated cardiorespiratory fitness.

^a^ Traditional risk factors include systolic blood pressure, diastolic blood pressure, antihypertensive medication, diabetes, total cholesterol/high-density lipoprotein cholesterol, lipid-lowering medication. Antihypertensive medication was excluded from the model using hypertension as an outcome; fasting blood glucose was included instead of diabetes in the model using diabetes as an outcome; estimated glomerular filtration rate at baseline was further included in the model using CKD as an outcome; prevalence of CVD was excluded from the model using CVD as an outcome.

## Discussion

### Principal Findings

In the present investigation, we observed several important findings. First, midlife eCRF was inversely associated with indices of arterial stiffness and subclinical atherosclerosis. Second, higher midlife eCRF is associated with a lower risk of developing hypertension, diabetes, CKD, CVD, and all-cause mortality over a mean follow-up of 15 years. Additionally, we observed a similar pattern of associations across all outcomes, with almost all eCRF measures. However, the strength of association observed using single occasion eCRF was relatively weaker compared with those using trajectories or average eCRF. Lastly, eCRF minimally improved the discriminative ability for CVD and all-cause mortality.

### Comparison With Previous Studies

#### Association of Midlife eCRF With Indices of Arterial Stiffness and Subclinical Atherosclerosis

Prior studies focused on CRF measured during in-person exercise testing and reported that CRF was inversely associated with CFPWV, brachial-ankle pulse wave velocity, and carotid atherosclerosis.^22,23,24,25,26,27^ Our investigation estimated CRF instead of measuring CRF and observed consistent associations. In particular, high midlife eCRF was associated with lower CFPWV and lower CIMT values.

We also observed an inverse relation of eCRF with prevalent coronary atherosclerosis, as demonstrated by CAC scores. Prior studies reported mixed results regarding the association between exercise-based CRF and coronary atherosclerosis. For example, several large cohort studies reported 30% to 40% lower odds of having CAC in individuals with high CRF when compared with individuals with low CRF.^28,29,30^ Our finding of an inverse linear relation between measures of eCRF and CAC is consistent with these investigations. However, other studies describe a U-shaped association between CRF and CAC.^31,32,33,34^ Although it is challenging to directly compare prior studies with our investigation—given the inherent differences in measuring CRF directly vs deriving eCRF indirectly using validated formulae and because of variable fitness among participants in different cohorts—the overall findings suggest that eCRF during midlife may serve as a marker of prevalent arterial stiffness and subclinical atherosclerosis.

#### Association of Midlife eCRF With the Incidence of Cardiometabolic Outcomes

Limited evidence exists regarding the associations between eCRF and incident cardiometabolic diseases. Our investigation provides insight into midlife eCRF and the relation with incident cardiometabolic diseases in later life. We observed that high eCRF, compared with low eCRF, was associated with a lower risk of developing hypertension, diabetes, and CKD, which is consistent with prior studies that used exercise-based CRF. For instance, individuals with high CRF have a 42% to 65% lower risk of developing hypertension over 5 to 9 years of follow-up compared with those with low CRF, and each MET increase in CRF was associated with a 10% to 19% lower risk of hypertension.^35,36,37,38,39,40,41,42^ High CRF in middle-aged adults has also been associated with a 39% to 67% lower risk of developing diabetes during 5 to 17 years of follow-up.^43,44,45,46,47,48,49,50,51,52,53^ Furthermore, CRF is associated with incident CKD, with studies demonstrating that highly fit individuals have a 27% to 58% lower risk of CKD compared with their less fit counterparts.^54,55,56^ Our findings support these aforementioned reports, suggesting that midlife eCRF may have a prognostic value for a range of cardiometabolic outcomes among middle-aged adults.

#### Association of Midlife eCRF With the Incidence of CVD and All-Cause Mortality

Few studies have examined the associations of eCRF with incident CVD and all-cause mortality. Thus far, it has been reported that the highest tertile of eCRF is associated with a 29% lower risk of coronary heart disease, compared with low eCRF,^57^ a 25% lower risk of acute MI,^58^ and 46% lower risk of stroke.^59^ Consistent with these findings, we observed a lower CVD risk in the most fit individuals (high eCRF) compared with less fit people (low eCRF). Additionally, a consistent inverse association between eCRF and mortality in middle-aged adults has been reported. In particular, investigators using data from the Third National Health and Nutrition Examination Survey cohort used the same eCRF algorithm as we did, and reported that middle-aged individuals in the highest eCRF tertile had a 44% lower risk of mortality compared with those with low eCRF, and that each MET increase in eCRF was associated with a lower mortality risk.^60^ Consistent with those findings, we observed a lower risk (using eCRF trajectories) of all-cause mortality in the most fit individuals compared with less fit participants. Further studies are warranted to assess the underlying mechanisms.

Lastly, large cohort studies have reported an improved discriminatory ability of eCRF for incident CVD and mortality when added to models including standard risk factors. In the present investigation, we observed a minimally improved discriminative ability of eCRF for CVD and mortality beyond that achieved by models including standard risk factors.

### Strengths and Limitations

There are several strengths of the present investigation. We used data from a large community-based sample, thus reducing selection bias. The use of eCRF also reduces selection bias because it is not dependent on a participant being healthy enough to complete an exercise test, but its estimation uses covariates that are routinely measured at serial FOS examinations. Additionally, residual confounding is minimized by using a well-characterized sample with a comprehensive assessment of CVD risk factors. We examined the associations of eCRF with a range of cardiometabolic disease outcomes and all-cause mortality using 3 different types of eCRF measures; this approach enables us to capture the entire midlife eCRF and evaluate its changes and associations with later life outcomes. Lastly, the eCRF algorithm used in the current investigation has been previously used to estimate CVD and all-cause mortality^60,61^

There are limitations of the present investigation that must be acknowledged. We modified the physical activity components of the nonexercise equation using the physical activity index, which may cause nondifferential misclassification; however, such misclassification tends to underestimate the strength of observed associations. Moreover, the eCRF algorithm has not been validated with exercise testing to measure CRF among FOS participants; it was validated using maximal treadmill testing in Aerobic Center Longitudinal Study participants, who have similar demographic characteristics to FOS participants (ie, mostly middle-aged, well-educated, non-Hispanic White individuals).^13,61^ Additionally, the accuracy of change in eCRF algorithms during midlife has not been fully evaluated. Investigators reported limited ability of eCRF algorithms to detect changes in directly measured CRF at 2 successive treadmill tests at least 3 months apart with a mean follow-up of 3 years^62^; however, our investigation calculated eCRF at 4 different time points between 1979 and 2001. Of note, incident CKD was defined based on single measurements of serum creatinine at each serial quadrennial FOS examination. Furthermore, our analysis did not adjust for alcohol consumption, dietary patterns, and socioeconomic status. Lastly, FOS is composed predominantly of White individuals of European ancestry, which limits the generalizability of our findings to other races or ethnicities. Thus, the observed associations of midlife eCRF with risk of cardiometabolic diseases and mortality should be confirmed in larger multiethnic cohorts.

## Conclusions

In this cohort study, higher midlife eCRF was associated with lower subclinical atherosclerosis and vascular stiffness and with a lower risk of hypertension, diabetes, chronic kidney disease, cardiovascular disease, and mortality. Our findings suggest that midlife eCRF may serve as a marker of cardiometabolic health and mortality in later life, highlighting the importance of adopting a healthy lifestyle, including regular physical activity during midlife.

## Notes

Supplement.
eMethods.

eReferences.
**eTable 1.** Characteristics of Participants Included and Excluded From the Analysis **eTable 2.** Age- and Sex-Specific Spearman Correlations Between Midlife eCRF and Its Components **eTable 3.** Association of Midlife eCRF With Subclinical Atherosclerosis in Participants Without CVD at Baseline **eTable 4.** Associations of midlife eCRF With the Incidence of Cardiometabolic Diseases and Mortality **eTable 5.** Associations of Midlife eCRF With the Incidence of Cardiometabolic Diseases and Mortality Among Participants Not on Antihypertensive Treatment **eTable 6.** Association of Midlife eCRF With the Incidence of Hypertension Based on the 2017 AHA/ACC Guidelines for Hypertension **eTable 7.** Associations of Midlife eCRF With Subclinical Atherosclerosis and the Incidence of Cardiometabolic Diseases and Mortality, Additionally Adjusting for eCRF Slope **eTable 8.** Associations of Midlife eCRF With the Incidence of Cardiometabolic Diseases and Mortality, Using eCRF Defined by Nes et al **eFigure.** Trajectories of Midlife eCRF
