# Supplementary material for: Association of Estimated Cardiorespiratory Fitness in Midlife With Cardiometabolic Outcomes and Mortality
Source: JAMA Netw Open. 2021 Oct 29;4(10):e2131284. doi: 10.1001/jamanetworkopen.2021.31284 (PMC8556623; doi:10.1001/jamanetworkopen.2021.31284)
Supplement: Supplement. — eMethods. eReferences. eTable 1. Characteristics of Participants Included and Excluded From the Analysis eTable 2. Age- and Sex-Specific Spearman Correlations Between Midlife eCRF and Its Components eTable 3. Association of Midlife eCRF With Subclinical Atherosclerosis in Participants Without CVD at Baseline eTable 4. Associations of midlife eCRF With the Incidence of Cardiometabolic Diseases and Mortality eTable 5. Associations of Midlife eCRF With the Incidence of Cardiometabolic Diseases and Mortality Among Participants Not on Antihypertensive Treatment eTable 6. Association of Midlife eCRF With the Incidence of Hypertension Based on the 2017 AHA/ACC Guidelines for Hypertension eTable 7. Associations of Midlife eCRF With Subclinical Atherosclerosis and the Incidence of Cardiometabolic Diseases and Mortality, Additionally Adjusting for eCRF Slope eTable 8. Associations of Midlife eCRF With the Incidence of Cardiometabolic Diseases and Mortality, Using eCRF Defined by Nes et al eFigure. Trajectories of Midlife eCRF [file jamanetwopen-e2131284-s001.pdf]

Supplemental Online Content

Lee J, Song RJ, Musa Yola I, et al. Association of estimated cardiorespiratory fitness in midlife with cardiometabolic outcomes and mortality. *JAMA Netw Open.* 2021;4(10):e2131284. doi:10.1001/jamanetworkopen.2021.31284

eMethods.

eReferences.

eTable 1. Characteristics of Participants Included and Excluded From the Analysis

eTable 2. Age- and Sex-Specific Spearman Correlations Between Midlife eCRF and Its Components

eTable 3. Association of Midlife eCRF With Subclinical Atherosclerosis in Participants Without CVD at Baseline

eTable 4. Associations of midlife eCRF With the Incidence of Cardiometabolic Diseases and Mortality

eTable 5. Associations of Midlife eCRF With the Incidence of Cardiometabolic Diseases and Mortality Among Participants Not on Antihypertensive Treatment

eTable 6. Association of Midlife eCRF With the Incidence of Hypertension Based on the 2017 AHA/ACC Guidelines for Hypertension

eTable 7. Associations of Midlife eCRF With Subclinical Atherosclerosis and the Incidence of Cardiometabolic Diseases and Mortality, Additionally Adjusting for eCRF Slope

eTable 8. Associations of Midlife eCRF With the Incidence of Cardiometabolic Diseases and Mortality, Using eCRF Defined by Nes et al

eFigure. Trajectories of Midlife eCRF

This supplemental material has been provided by the authors to give readers additional information about their work.

© 2021 Lee J et al. *JAMA Network Open*.

eMETHODS. Detailed methods

Study sample

Overall, analyses were conducted based on two large subsamples of the Framingham Offspring Study (FOS). First, 3,539 participants who attended the seventh examination cycle (1998-2001) were eligible (**Sample 1.1** – **Sample 1.8**). Of these 3,539 participants, we excluded those aged beyond the limits for which estimated cardiorespiratory fitness (eCRF) algorithms were developed (<20 years of age, as well as, women >76 and men >86 years of age), body mass index (BMI) ≤18.5 kg/m^2, and/or those with unavailable data for calculation eCRF and covariates at baseline (n=577) to examine the association of eCRF at the seventh examination ($eCRF_{exam7}$) with all-cause mortality (**Sample 1.1**; n=2,962). To investigate the relation of $eCRF_{exam7}$ with incident cardiometabolic diseases (hypertension, diabetes, chronic kidney disease [CKD], and cardiovascular disease [CVD]), we then excluded participants with prevalent cardiometabolic disease or unavailable data on cardiometabolic disease status at the seventh examination, resulting in final sample sizes of 1,506 (**Sample 1.2**; hypertension), 2,268 (**Sample 1.3**; diabetes), 2,343 (**Sample 1.4**; CKD), and 2,608 (**Sample 1.5**; CVD), respectively. We examined the cross-sectional associations of $eCRF_{exam7}$ with carotid-femoral pulse wave velocity (CFPWV, **Sample 1.6**; n=2,041), carotid intima-media thickness (CIMT, **Sample 1.7**; n=2,106), and coronary artery calcium score (CAC, **Sample 1.8**; n=1,172).

Additionally, 2,820 participants who attended the second (1979-1983), fourth (1987-1991), fifth (1991-1995), and seventh (1998-2001) examination cycles were eligible to examine associations of the trajectories of eCRF ($eCRF_{trajectories}$) and the standardized average of eCRF ($eCRF_{average}$) between second and seventh examinations with outcomes (**Sample 2.1** – **Sample 2.8**). After excluding those aged beyond the range for which eCRF algorithms were developed, BMI ≤18.5 kg/m^2, and/or with unavailable data for eCRF calculation, outcomes, and covariates

© 2021 Lee J et al. *JAMA Network Open*.

at the seventh examination, we examined the associations of midlife $eCRF_{trajectories}$ and $eCRF_{average}$ with the incidence of all-cause mortality (**Sample 2.1**; n=2,247), hypertension (**Sample 2.2**; n=1,159), diabetes (**Sample 2.3**; n=1,770), CKD (**Sample 2.4**; n=1,817), and CVD (**Sample 2.5**; n=1,970). The cross-sectional associations of $eCRF_{trajectories}$ and $eCRF_{average}$ with prevalent subclinical atherosclerosis were examined using subsamples of Sample 2.1 (CFPWV [**Sample 2.6**; n=1,498], CIMT [**Sample 2.7**; n=1,699], and CAC score [**Sample 2.8**; n=970]).

eCRF assessment

During each Heart Study examination, data for eCRF calculations were obtained. Data on age and sex were collected using a medical history questionnaire. Height and weight were measured via a standardized scale, and BMI was calculated as the weight in kilograms divided by the square of the height in meters. Waist circumference was measured by a trained professional applying anthropometric tape at the level of the umbilicus and recording the reading at mid-respiration and rounding to the nearest 0.25 of an inch. Resting heart rate was measured using standard supine 12-lead electrocardiography performed after approximately 5 minutes of resting quietly. Physical activity was measured using the Physical Activity Index (PAI), which measures self-reported intensity, frequency, and duration of physical activity during leisure time and is categorized as active (yes) or insufficiently active (no) based on the median values of PAI as previously described.[1] Participants who self-reported smoking ≥1 cigarette/day during the year before each examination cycle were classified as current smokers.

© 2021 Lee J et al. *JAMA Network Open*.

Indicators of arterial stiffness and subclinical atherosclerosis

To quantify CFPWV, applanation tonometry was performed after participants rested for 5 minutes in the supine position using a commercially available tonometer (SPT-301, Millar Instruments, Houston, TX).[2] For each participant, the transit distances from the suprasternal notch to carotid and femoral sites were measured, and the difference between the two lengths was divided by the delay in time between the bottom of the carotid and femoral waveforms, as previously described.[3] All measures were stored digitally in a core (Cardiovascular Engineering, Inc, Norwood, MA) and blinded to clinical information for subsequent analyses.[2]

Standard coronary computed tomography angiography was performed to calculate the standardized CAC score in which a *calcified lesion* was defined per the following standards: an area ≥3 connected pixels, an attenuation >130 Hounsfield units. An Agatston score was calculated as previously described.[4] Results were evaluated by a certified clinician.

Lastly, ultrasound of the carotid arteries was performed during the eighth examination cycle to quantify CIMT. The common carotid arteries (CCA) were imaged with a 7.5-MHz transducer while the carotid bulb and internal carotid arteries (ICA) were imaged with a 5-MHz transducer (3-dB point: 6.2 MHz). Color Doppler and Doppler spectral analyzer software (model SSH140A; Toshiba America Medical Systems) were applied.[5,6] Gated diastolic images were obtained of the left and right carotid arteries at the level of the distal CCA, the carotid artery bulb, and the proximal 2 cm of the ICA. Assessment by two independent interpreters (replicate readings, n=25) for the mean ICA and CCA IMT showed intraclass correlation coefficients of 0.74 and 0.90, respectively.[7]

© 2021 Lee J et al. *JAMA Network Open*.

Covariates

Baseline blood pressure was measured twice at rest in a seated position by a physician using a mercury column sphygmomanometer and measurements were averaged. Fasting plasma glucose, total cholesterol (TC), and high-density lipoprotein cholesterol (HDL-C) concentrations were measured using standard enzymatic methods. Serum creatinine was measured using the modified Jaffé method and adjusted by a two-step serum creatinine calibration process as previously described.[8] The CKD-Epidemiology Collaboration (CKD-EPI) equation was used to estimate the glomerular filtration rate (eGFR).[9] Information on cardiovascular disease was collected by a medical history questionnaire, physical examination, and hospitalization records.

Statistical analysis

For analyses of eCRF trajectories, we limited the number of trajectory groups to ≤5, using the Bayesian Information Criterion to assess the best model fit. We accounted for the uncertainty of eCRF group membership by simulating 50 imputed datasets of trajectory group assignment re-classifying participants using the participant's posterior group probability. We then combined estimates from the 50 datasets using PROC MIANALYZE.

© 2021 Lee J et al. *JAMA Network Open*.

eReferences

1. Kannel WB, Sorlie P. Some health benefits of physical activity. The Framingham Study. *Arch Intern Med.* 1979;139(8):857-861.

2. Mitchell GF, Parise H, Benjamin EJ, et al. Changes in arterial stiffness and wave reflection with advancing age in healthy men and women: the Framingham Heart Study. *Hypertension.* 2004;43(6):1239-1245.

3. Mitchell GF, Izzo JL, Jr., Lacourciere Y, et al. Omapatrilat reduces pulse pressure and proximal aortic stiffness in patients with systolic hypertension: results of the conduit hemodynamics of omapatrilat international research study. *Circulation.* 2002;105(25):2955-2961.

4. Parikh NI, Hwang SJ, Larson MG, et al. Parental occurrence of premature cardiovascular disease predicts increased coronary artery and abdominal aortic calcification in the Framingham Offspring and Third Generation cohorts. *Circulation.* 2007;116(13):1473-1481.

5. O'Leary DH, Polak JF, Kronmal RA, et al. Distribution and correlates of sonographically detected carotid artery disease in the Cardiovascular Health Study. The CHS Collaborative Research Group. *Stroke.* 1992;23(12):1752-1760.

6. Polak JF, O'Leary DH, Kronmal RA, et al. Sonographic evaluation of carotid artery atherosclerosis in the elderly: relationship of disease severity to stroke and transient ischemic attack. *Radiology.* 1993;188(2):363-370.

7. Ellis K, Pothier CE, Blackstone EH, Lauer MS. Is systolic blood pressure recovery after exercise a predictor of mortality? *Am Heart J.* 2004;147(2):287-292.

8. Coresh J, Astor BC, McQuillan G, et al. Calibration and random variation of the serum creatinine assay as critical elements of using equations to estimate glomerular filtration rate. *Am J Kidney Dis.* 2002;39(5):920-929.

9. Levey AS, Stevens LA, Schmid CH, et al. A new equation to estimate glomerular filtration rate. *Ann Intern Med.* 2009;150(9):604-612.

© 2021 Lee J et al. *JAMA Network Open*.

eTable 1. Characteristics of participants included and excluded from the analysis.

Variable	Included (n=2,962)	Excluded (n=577)
Age, years	61 (9)	66 (11)
Women, n (%)	1562 (52.7)	352 (61.0)
Body mass index, kg/m^2	28.2 (5.2)	27.7 (6.2)
Waist circumference, cm	100.1 (14.0)	98.3 (15.0)
Resting heart rate, beats/minute	65 (11)	67 (12)
Physical activity index	38 (6.4)	35.7 (5.1)
SBP, mmHg	127 (19)	131 (20)
DBP, mmHg	74 (10)	73 (11)
Hypertension, n (%)	1308 (44.2)	326 (57.6)
Antihypertensive medication, n (%)	972 (32.8)	246 (43.4)
Fasting glucose, mg/dL	104 (25)	109 (36)
Diabetes, n (%)	320 (10.8)	75 (19.6)
Diabetes medication, n (%)	188 (6.4)	59 (10.3)
Total cholesterol, mg/dL	200 (37)	200 (39)
HDL-C, mg/dL	54 (17)	55 (19)
Lipid-lowering medication, n (%)	603 (20.4)	139 (24.2)
eGFR, $ml/min/1.73m^2$	83.8 (16.3	79 (19)
Internal carotid artery IMT, mm	2.3 (1.1)	2.5 (1.2)
Common carotid artery IMT, mm	0.7 (0.2)	0.8 (0.2)
CFPWV, m/s	10.0 (3.4)	11.4 (4.5)
CAC, AU, median (Q1, Q3)	34.1 (0, 237.7)	81.7 (1.4, 316.2)
Prevalent CKD, n (%)	242 (8.2)	53 (14.9)
Prevalent CVD, n (%)	354 (12.0)	125 (21.7)
eCRF, METs	9.3 (2.0)	7.7 (2.4)

Abbreviations: SBP, systolic blood pressure; DBP, diastolic blood pressure; HDL-C, high-density lipoprotein cholesterol; eGFR, estimated glomerular filtration; IMT, intima-media thickness; CFPWV, carotid-femoral pulse wave velocity; CAC, coronary artery calcium; AU, Agatston unit; CKD, chronic kidney disease; CVD, cardiovascular disease; eCRF, non-exercise estimated cardiorespiratory fitness; METs, metabolic equivalent of tasks.

Note: Values are mean (standard deviation) unless otherwise indicated.

© 2021 Lee J et al. *JAMA Network Open*.

eTable 2. Age- and sex-specific Spearman correlations between midlife eCRF and its components

**$eCRF_{exam7}$ (N=2,692)**	$eCRF_{exam7}$	BMI	WC	Resting HR	PAI	Smoking
$eCRF_{exam7}$	1	-0.85*	-0.82*	-0.46*	0.28*	-0.08*
BMI		1	0.88*	0.19*	-0.08*	-0.03
WC			1	0.19*	-0.13*	-0.01
Resting HR				1	-0.06†	0.10*
PAI					1	0.01
Smoking						1
**$eCRF_{trajectories}$ (N=2,247)**	$eCRF_{trajectories}$	BMI	WC	Resting HR	PAI	Smoking
$eCRF_{trajectories}$	1	-0.72*	0.70*	-0.32*	0.14*	-0.07†
BMI		1	0.88*	0.19*	-0.06†	-0.02
WC			1	0.19*	-0.12*	0.003
Resting HR				1	-0.06†	0.09*
PAI					1	-0.01
Smoking						1
**$eCRF_{average}$ (N=2,247)**	$eCRF_{average}$	BMI	WC	Resting HR	PAI	Smoking
$eCRF_{average}$	1	-0.81*	-0.78*	-0.36*	0.16*	-0.09*
BMI		1	0.88*	0.19*	-0.06†	-0.02
WC			1	0.19*	-0.12*	-0.003
Resting HR				1	-0.06†	0.09*
PAI					1	-0.03
Smoking						1

Abbreviations: eCRF, non-exercise estimated cardiorespiratory fitness; BMI, body mass index; WC, waist circumference; HR, heart rate; PAI, physical activity index.

Note: Sex-specific eCRF at the seventh examinations were converted into z-score with mean of 0 and standard deviation of 1 ($eCRF_{exam7}$); Sex-specific eCRF trajectories between second and seventh examinations were determined using group based modeling strategy (SAS PROC TRAJ, $eCRF_{trajectories}$); Sex-specific average of eCRF between second and seventh examinations were converted into z-score with mean of 0 and standard deviation of 1 ($eCRF_{average}$).

*P<0.001; †P<0.01; ‡P<0.05.

© 2021 Lee J et al. *JAMA Network Open*.

eTable 3. Association of midlife eCRF with subclinical atherosclerosis in participants without CVD at baseline.

**-1000/CFPWV (ms/m)**	$eCRF_{exam7}$ (n=1,792)				$eCRF_{trajectories}$ (n=1,304)				$eCRF_{average}$ (n=1,304)			
	N	β est.	SE	P value	N	β est.	SE	P value	N	β est.	SE	P value
eCRF (per 1 SD increment)	1,792	-5.84	0.63	**<.001**	1,304	--	--	--	1,498	-5.51	0.74	**<.001**
Low eCRF	458	Referent			153	Referent			427	Referent		
Moderate eCRF	618	-6.37	1.18	**<.001**	598	-6.60	1.75	**<.001**	521	-6.00	1.40	**<.001**
High eCRF	716	-12.27	1.41	**<.001**	553	-13.08	2.05	**<.001**	550	-10.55	1.65	**<.001**
**CIMT (mm)**	$eCRF_{exam7}$ (n=1,889)				$eCRF_{trajectories}$ (n=1,518)				$eCRF_{average}$ (n=1,518)			
	N	β est.	SE	P value	N	β est.	SE	P value	N	β est.	SE	P value
eCRF (per 1 SD increment)	1,889	-0.05	0.02	**<.001**	1,518	--	--	--	1,518	-0.07	0.02	**<.001**
Low eCRF	510	Referent			205	Referent			407	Referent		
Moderate eCRF	657	-0.03	0.04	.44	680	-0.11	0.06	.06	518	-0.05	0.05	.24
High eCRF	722	-0.10	0.05	**.03**	633	-0.18	0.06	**.005**	593	-0.13	0.05	**.02**
**CAC (AU)**	$eCRF_{exam7}$ (n=1,076)				$eCRF_{trajectories}$ (n=886)				$eCRF_{average}$ (n=886)			
	N	β est.	SE	P value	N	β est.	SE	P value	N	β est.	SE	P value
eCRF (per 1 SD increment)	1,076	-0.16	0.09	**.04**	886	--	--	--	886	-0.28	0.09	**.02**
Low eCRF	282	Referent			104	Referent			211	Referent		
Moderate eCRF	381	-0.04	0.17	.81	388	-0.19	0.25	.45	308	-0.32	0.19	.09
High eCRF	413	-0.27	0.20	.17	394	-0.65	0.27	**.02**	367	-0.65	0.21	**.002**

Abbreviations: eCRF, non-exercise estimated cardiorespiratory fitness; CVD, cardiovascular disease; CFPWV, carotid femoral pulse wave velocity; est., estimate; SE, standard error; SD, standard deviation; CIMT, carotid intima-media thickness; CAC, coronary artery calcium; AU, Agatston unit.
Note: Models were adjusted for age, sex, systolic blood pressure, diastolic blood pressure, antihypertensive medication, diabetes, total cholesterol/high-density lipoprotein cholesterol, and lipid-lowering medication at exam 7; SDs are equal to 2.0 MET for all models.

© 2021 Lee J et al. *JAMA Network Open*.

eTable 4. Associations of midlife eCRF with the incidence of cardiometabolic diseases and mortality.

**Hypertension**	eCRF$_{exam7}$ (n=1,506)			eCRF$_{trajectories}$ (n=1,159)			eCRF$_{average}$ (n=1,159)		
	#events/ #at risk	HR (95% CI)	P value	#events/ #at risk	HR (95%CI)	P value	#events/ #at risk	HR (95% CI)	P value
eCRF (per 1 SD increment)	728/1,506	0.82 (0.72-0.94)	**.004**	580/1,159	--	--	580/1,159	0.74 (0.64-0.86)	**<.001**
Low eCRF	196/292	Referent		67/100	Referent		147/212	Referent	
Moderate eCRF	273/488	0.83 (0.63-1.09)	.18	282/451	0.81 (0.53-1.26)	.35	224/381	0.69 (0.50-0.94)	**.02**
High eCRF	259/726	0.63 (0.46-0.85)	**.003**	231/608	0.54 (0.34-0.87)	**.01**	209/566	0.48 (0.34-0.68)	**<.001**
**Diabetes**	eCRF$_{exam7}$ (n=2,268)			eCRF$_{trajectories}$ (n=1,770)			eCRF$_{average}$ (n=1,770)		
	# events/ #at risk	HR (95% CI)	P value	# events/ #at risk	HR (HR-95% CI)	P value	# events/ #at risk	HR (95% CI)	P value
eCRF (per 1 SD increment)	214/2,268	0.67 (0.56-0.82)	**<.001**	169/1,770	--	--	169/1,770	0.64 (0.52-0.79)	**<.001**
Low eCRF	95/573	Referent		55/206	Referent		86/443	Referent	
Moderate eCRF	89/801	0.74 (0.52-1.04)	.08	86/807	0.46 (0.29-0.72)	**<.001**	57/617	0.51 (0.34-0.76)	**.001**
High eCRF	30/894	0.38 (0.23-0.62)	**<.001**	28/757	0.27 (0.15-0.48)	**<.001**	26/710	0.31 (0.18-0.54)	**<.001**
**CKD**	eCRF$_{exam7}$ (n=2,343)			eCRF$_{trajectories}$ (n=1,817)			eCRF$_{average}$ (n=1,817)		
	# events/ #at risk	HR (95% CI)	P value	# events/ #at risk	HR (HR-95% CI)	P value	# events/ #at risk	HR (95% CI)	P value
eCRF (per 1 SD increment)	439/2,343	0.87 (0.75-1.01)	.07	358/1,817	--	--	358/1,817	0.84 (0.72-0.98)	**.03**
Low eCRF	189/632	Referent		81/249	Referent		147/484	Referent	
Moderate eCRF	150/810	0.69 (0.53-0.89)	**.005**	192/815	0.68 (0.47-0.98)	**.04**	134/629	0.75 (0.56-1.00)	**.049**
High eCRF	100/901	0.89 (0.63-1.25)	.49	85/753	0.63 (0.40-0.97)	**.04**	77/704	0.64 (0.44-0.94)	**.02**
**CVD**	eCRF$_{exam7}$ (n=2,608)			eCRF$_{trajectories}$ (n=1,970)			eCRF$_{average}$ (n=1,970)		
	# events/ #at risk	HR (95% CI)	P value	# events/ #at risk	HR (HR-95% CI)	P value	# events / #at risk	HR (95% CI)	P value
eCRF (per 1 SD increment)	500/2,608	0.83 (0.75-0.93)	**<.001**	392/1,970	--	--	392/1,970	0.75 (0.67-0.84)	**<.001**
Low eCRF	230/784	Referent		101/298	Referent		195/583	Referent	
Moderate eCRF	176/882	0.85 (0.69-1.04)	.12	227/900	0.87 (0.66-1.14)	.30	137/667	0.75 (0.59-0.94)	**.01**
High eCRF	94/942	0.71 (0.53-0.95)	**.02**	64/772	0.46 (0.31-0.68)	**<.001**	60/720	0.43 (0.30-0.60)	**<.001**
**All-cause mortality**	eCRF$_{exam7}$ (n=2,962)			eCRF$_{trajectories}$ (n=2,247)			eCRF$_{average}$ (n=2,247)		

© 2021 Lee J et al. *JAMA Network Open*.

	# events / #at risk	HR (95% CI)	P value	# events / #at risk	HR (HR-95% CI)	P value	# events / #at risk	HR (95% CI)	P value
eCRF (per 1 SD increment)	770/2,962	0.84 (0.77-0.92)	**<.001**	600/2,247	--	--	600/2,247	0.81 (0.73-0.89)	**<.001**
Low eCRF	404/961	Referent		201/384	Referent		316/727	Referent	
Moderate eCRF	240/1,003	0.82 (0.69-0.97)	**.02**	290/1,046	0.67 (0.56-0.82)	**<.001**	182/757	0.77 (0.64-0.94)	**.009**
High eCRF	126/998	0.83 (0.65-1.06)	.13	109/817	0.69 (0.50-0.95)	**.03**	102/763	0.80 (0.61-1.05)	.11

Abbreviations: eCRF, non-exercise estimated cardiorespiratory fitness; HR, hazard ratio; CI, confidence interval; SD, standard deviation; CKD, chronic kidney disease; CVD, cardiovascular disease.

Note: Models were adjusted for age, sex, systolic blood pressure, diastolic blood pressure, antihypertensive medication, diabetes, total cholesterol/high-density lipoprotein cholesterol, lipid-lowering medication, and prevalence of CVD; Antihypertensive medication was not adjusted in the model using hypertension as an outcome; Fasting blood glucose was adjusted instead of diabetes in the model using diabetes as an outcome; estimated glomerular filtration rate was further adjusted in the model using CKD as an outcome; Prevalence of CVD was excluded in the model using CVD as an outcome; SDs are equal to 1.9 MET for hypertension, diabetes, and CKD samples and 2.0 MET for CVD and all-cause mortality sample.

© 2021 Lee J et al. *JAMA Network Open*.

eTable 5. Associations of midlife eCRF with the incidence of cardiometabolic diseases and mortality among participants not on antihypertensive treatment.

**Hypertension**	$eCRF_{exam7}$ (n=1,506)			$eCRF_{trajectories}$ (n=1,159)			$eCRF_{average}$ (n=1,159)		
	#events/ #at risk	HR (95% CI)	P value	#events/ #at risk	HR (95%CI)	P value	#events/ #at risk	HR (95% CI)	P value
eCRF (per 1 SD increment)	--	--	**--**	**--**	--	--	-	--	**--**
Low eCRF	--	--	--	--	--	--	--	--	--
Moderate eCRF	--	--	--	--	--	--	--	--	**--**
High eCRF	--	--	**--**	--	--	**--**	--	--	**--**
**Diabetes**	$eCRF_{exam7}$ (n=1,506)			$eCRF_{trajectories}$ (n=1,159)			$eCRF_{average}$ (n=1,159)		
	# events/ #at risk	HR (95% CI)	P value	# events/ #at risk	HR (HR-95% CI)	P value	# events/ #at risk	HR (95% CI)	P value
eCRF (per 1 SD increment)	120/1,657	0.72 (0.55-0.93)	**.01**	93/1,281	--	--	93/1,281	0.70 (0.53-0.93)	**.01**
Low eCRF	46/332	Referent		22/115	Referent		36/244	Referent	
Moderate eCRF	50/561	0.69 (0.43-1.14)	.15	47/511	0.59 (0.31-1.11)	.10	36/421	0.59 (0.34-1.04)	.07
High eCRF	24/764	0.44 (0.23-0.84)	**.01**	24/655	0.36 (0.17-0.77)	**.008**	21/616	0.36 (0.18-0.72)	**.004**
**CKD**	$eCRF_{exam7}$ (n=1,506)			$eCRF_{trajectories}$ (n=1,159)			$eCRF_{average}$ (n=1,159)		
	# events/ #at risk	HR (95% CI)	P value	# events/ #at risk	HR (HR-95% CI)	P value	# events/ #at risk	HR (95% CI)	P value
eCRF (per 1 SD increment)	254/1,686	0.91 (0.75-1.11)	.36	210/1,299	--	--	210/1,299	0.86 (0.69-1.06)	.15
Low eCRF	92/353	Referent		36/129	Referent		71/258	Referent	
Moderate eCRF	85/562	0.67 (0.46-0.95)	**.03**	108/516	0.72 (0.44-1.19)	.20	78/427	0.68 (0.46-1.01)	.06
High eCRF	77/771	0.92 (0.60-1.43)	.71	66/654	0.65 (0.36-1.15)	.14	61/614	0.60 (0.37-0.96)	**.03**
**CVD**	$eCRF_{exam7}$ (n=1,506)			$eCRF_{trajectories}$ (n=1,159)			$eCRF_{average}$ (n=1,159)		
	# events/ #at risk	HR (95% CI)	P value	# events/ #at risk	HR (HR-95% CI)	P value	# events / #at risk	HR (95% CI)	P value
eCRF (per 1 SD increment)	293/1,865	0.85 (0.73-0.97)	**.02**	229/1,402	--	--	229/1,402	0.72 (0.62-0.84)	**<.001**
Low eCRF	120/443	Referent		49/157	Referent		96/314	Referent	
Moderate eCRF	100/606	0.86 (0.65-1.14)	.29	132/570	0.84 (0.59-1.21)	.36	87/454	0.75 (0.55-1.01)	.06
High eCRF	73/816	0.73 (0.51-1.04)	.08	48/675	0.43 (0.26-0.69)	**<.001**	46/634	0.40 (0.27-0.61)	**<.001**

© 2021 Lee J et al. *JAMA Network Open*.

**All-cause mortality**	eCRF$_{exam7}$ (n=1,506)			eCRF$_{trajectories}$ (n=1,159)			eCRF$_{average}$ (n=1,159)		
	# events / #at risk	HR (95% CI)	P value	# events / #at risk	HR (HR-95% CI)	P value	# events / #at risk	HR (95% CI)	P value
eCRF (per 1 SD increment)	397/1,990	0.85 (0.75-0.97)	**.02**	311/1,494	--	--	311/1,494	0.79 (0.69-0.91)	**.001**
Low eCRF	181/498	Referent		82/182	Referent		133/349	Referent	
Moderate eCRF	127/651	0.82 (0.64-1.04)	.11	148/615	0.64 (0.48-0.86)	**.003**	101/490	0.71 (0.54-0.93)	**.01**
High eCRF	89/841	0.82 (0.60-1.13)	.23	81/697	0.66 (0.44-0.99)	**.047**	77/655	0.75 (0.54-1.06)	.11

Abbreviations: eCRF, non-exercise estimated cardiorespiratory fitness; HR, hazard ratio; CI, confidence interval; SD, standard deviation; CKD, chronic kidney disease; CVD, cardiovascular disease.

Note: Models were adjusted for age, sex, systolic blood pressure, diastolic blood pressure, antihypertensive medication, diabetes, total cholesterol/high-density lipoprotein cholesterol, lipid-lowering medication, and prevalence of CVD; No results were reported regarding the relation between eCRF and incident hypertension because the definition of hypertension include patients on antihypertensive treatment; Fasting blood glucose was adjusted instead of diabetes in the model using diabetes as an outcome; Estimated glomerular filtration rate was further adjusted in the model using CKD as an outcome; Prevalence of CVD was excluded in the model using CVD as an outcome; SDs are equal to 1.9 MET for diabetes and CKD samples and 2.0 MET for CVD and all-cause mortality sample.

© 2021 Lee J et al. *JAMA Network Open*.

eTable 6. Association of midlife eCRF with the incidence of hypertension based on the 2017 AHA/ACC guidelines for hypertension.

**Hypertension**	eCRF$_{exam7}$ (n=1,076)			eCRF$_{trajectories}$ (n=832)			eCRF$_{average}$ (n=832)		
	#events/ #at risk	HR (95% CI)	P value	#events/ #at risk	HR (95%CI)	P value	#events/ #at risk	HR (95% CI)	P value
eCRF (per 1 SD increment)	630/1,076	0.85 (0.72-1.01)	.06	495/832	--	--	495/832	0.78 (0.65-0.93)	**.006**
Low eCRF	128/175	Referent		42/61	Referent		98/135	Referent	
Moderate eCRF	221/334	0.93 (0.65-1.31)	.66	207/300	1.12 (0.65-1.92)	.68	170/254	0.81 (0.55-1.19)	.28
High eCRF	281/567	0.75 (0.51-1.09)	.13	246/471	0.85 (0.49-1.49)	.57	227/443	0.68 (0.46-1.03)	.07

Abbreviations: eCRF, non-exercise estimated cardiorespiratory fitness; AHA, American Heart Association; ACC, American College of Cardiology; HR, hazard ratio; CI, confidence interval; SD, standard deviation

Note: Models were adjusted for age, sex, systolic blood pressure, diastolic blood pressure, diabetes, total cholesterol/high-density lipoprotein cholesterol, lipid-lowering medication, and prevalence of CVD.

© 2021 Lee J et al. *JAMA Network Open*.

eTable 7. Associations of midlife eCRF with subclinical atherosclerosis and the incidence of cardiometabolic diseases and mortality, additionally adjusting for eCRF slope.

**-1000/CFPWV (ms/m)**	$eCRF_{exam7}$ (n=2,041)			
	N	β est.	SE	P value
eCRF (per 1 SD increment)	2,041	-4.90	0.70	**<.001**
**CIMT (mm)**	$eCRF_{exam7}$ (n=2,106)			
	N	β est.	SE	P value
eCRF (per 1 SD increment)	2,106	-0.08	0.02	**<.001**
**CAC (AU)**	$eCRF_{exam7}$ (n=1,172)			
	N	β est.	SE	P value
eCRF (per 1 SD increment)	1,172	-0.34	0.09	0.20

**Hypertension**	$eCRF_{exam7}$ (n=1,506)		
	#events/ #at risk	HR (95% CI)	P value
eCRF (per 1 SD increment)	728/1,506	0.72 (0.61-0.84)	**<.001**
**Diabetes**	$eCRF_{exam7}$ (n=2,268)		
	# events/ #at risk	HR (95% CI)	P value
eCRF (per 1 SD increment)	214/2,268	0.67 (0.53-0.82)	**<.001**
**CKD**	$eCRF_{exam7}$ (n=2,343)		
	# events/ #at risk	HR (95% CI)	P value
eCRF (per 1 SD increment)	439/2,343	0.81 (0.69-0.95)	**.011**
**CVD**	$eCRF_{exam7}$ (n=2,608)		
	# events/ #at risk	HR (95% CI)	P value
eCRF (per 1 SD increment)	500/2,608	0.73 (0.65-0.82)	**<.001**
**All-cause mortality**	$eCRF_{exam7}$ (n=2,962)		
	# events / #at risk	HR (95% CI)	P value
eCRF (per 1 SD increment)	770/2,962	0.80 (0.72-0.89)	**<.001**

Abbreviations: eCRF, non-exercise estimated cardiorespiratory fitness; HR, hazard ratio; CI, confidence interval; SD, standard deviation; CIMT, carotid intima-media thickness; CAC, coronary artery calcium; AU, Agatston units; CKD, chronic kidney disease; CVD, cardiovascular disease.

Note: Models were adjusted for age, sex, systolic blood pressure, diastolic blood pressure, antihypertensive medication, diabetes, total cholesterol/high-density lipoprotein cholesterol, lipid-lowering medication, and prevalence of CVD; Antihypertensive medication was not adjusted in the model using hypertension as an outcome; Fasting blood glucose was adjusted instead of diabetes in the model using diabetes as an outcome; estimated glomerular filtration rate was further adjusted in the model using CKD as an outcome; Prevalence of CVD was excluded in the model using CVD as an outcome; SDs are equal to 1.9 MET for hypertension, diabetes, and CKD samples and 2.0 MET for CVD and all-cause mortality sample.

© 2021 Lee J et al. *JAMA Network Open*.

eTable 8. Associations of midlife eCRF with incidence of cardiometabolic diseases and mortality, using eCRF defined by Nes et al.

**Hypertension**	$eCRF_{exam7}$ (n=1,506)		
	#events/ #at risk	HR (95% CI)	P value
eCRF, original (per 1 SD increment)	728/1,506	0.82 (0.72-0.94)	**.004**
eCRF, Nes et al. (per 1 SD increment)	728/1,506	0.84 (0.74-0.95)	**.006**
**Diabetes**	$eCRF_{exam7}$ (n=2,268)		
	# events/ #at risk	HR (95% CI)	P value
eCRF, original (per 1 SD increment)	214/2,268	0.67 (0.56-0.82)	**<.001**
eCRF, Nes et al. (per 1 SD increment)	214/2,268	0.68 (0.57-0.81)	**<.001**
**CKD**	$eCRF_{exam7}$ (n=2,343)		
	# events/ #at risk	HR (95% CI)	P value
eCRF, original (per 1 SD increment)	439/2,343	0.87 (0.75-1.01)	.07
eCRF, Nes et al. (per 1 SD increment)	439/2,343	0.90 (0.78-1.03)	.11
**CVD**	$eCRF_{exam7}$ (n=2,608)		
	# events/ #at risk	HR (95% CI)	P value
eCRF, original (per 1 SD increment)	500/2,608	0.83 (0.75-0.93)	**<.001**
eCRF, Nes et al. (per 1 SD increment)	500/2,608	0.86 (0.78-0.95)	**.004**
**All-cause mortality**	$eCRF_{exam7}$ (n=2,962)		
	# events / #at risk	HR (95% CI)	P value
eCRF, original (per 1 SD increment)	770/2,962	0.84 (0.77-0.92)	**<.001**
eCRF, Nes et al. (per 1 SD increment)	770/2,962	0.92 (0.84-0.996)	**.040**

© 2021 Lee J et al. *JAMA Network Open*.

eFigure. Trajectories of midlife eCRF.

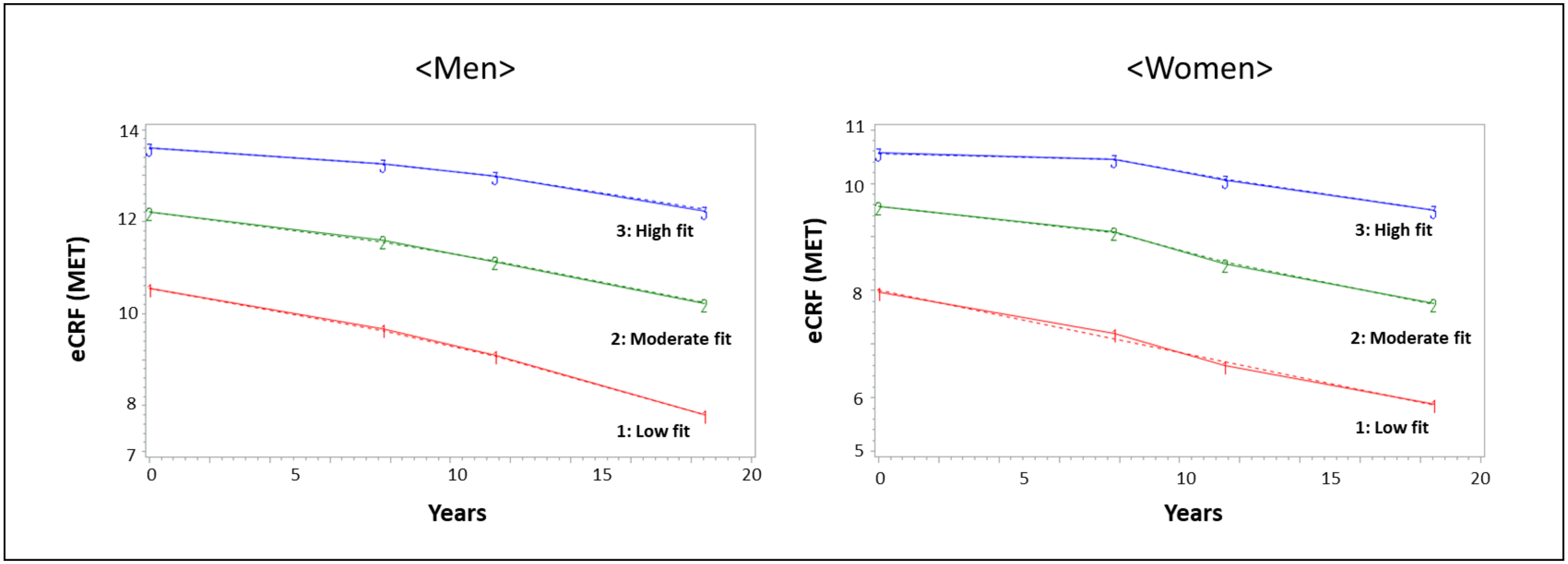

Abbreviations: eCRF, non-exercise estimated cardiorespiratory fitness; MET, metabolic equivalent of task.

© 2021 Lee J et al. *JAMA Network Open*.
